# Prevalence of pre- and postpartum depression in Jamaican women

**DOI:** 10.1186/1471-2393-5-15

**Published:** 2005-11-08

**Authors:** Janice Wissart, Omkar Parshad, Santosh Kulkarni

**Affiliations:** 1 Department of Basic Medical Sciences, Faculty of Medical Sciences, The University of the West Indies, Mona, Kingston 7, Jamaica (WI); 2 Department of Obstetrics, Gynaecology and Child Health, Faculty of Medical Sciences, The University of the West Indies, Mona, Kingston 7, Jamaica (WI)

## Abstract

**Background:**

Maternal depression during pregnancy has been studied less than depression in postpartum period. The aims of this study were to find out the prevalence of prepartum and postpartum depression and the risk factors associated in a cohort of Afro-Jamaican pregnant women in Jamaica.

**Methods:**

The Zung self-rating depression scale instrument was administered to 73 healthy pregnant women at 28 weeks gestation and at 6 weeks postpartum for quantitative measurement of depression. Blood samples were collected at 8, 28, 35 weeks gestation and at day 1 and 6 weeks postpartum to study the thyroid status.

**Results:**

Study demonstrated depression prevalence rates of 56% and 34% during prepartum and postpartum period, respectively. 94% women suffering depression in both periods were single. There were significant variations in both FT_3 _and TT_4 _concentrations which increased from week 8 to week 28 prepartum (p < 0.05) and then declined at the 35^th ^week (p < 0.05 compared with week 28) and 1 day post delivery study (p < 0.05 compared with week 35). The mean values for TSH increased significantly from week 8 through week 35. The mean values at 1 day postpartum and 6 week postpartum were not significantly different from the 35 week values. For FT_3_, TT_4 _and TSH there were no significant between group differences in concentrations. The major determinants of postpartum depression were moderate and severe prepartum depression and change in TT_4 _hormone concentrations.

**Conclusion:**

High prevalence of depression was found during pre- and postpartum periods. Single mothers, prepartum depression and changes in TT_4 _were factors found to be significantly associated with postpartum depression.

## Background

Maternal depression during pregnancy has been studied less than depression in postpartum period. A recent study, however, shows that depression seems to be as common during pregnancy as after delivery [1]. Depending upon various scales used to measure depression, the prevalence rate has been found to be between 5% and 26% for antenatal depression [2-6] and between 7% and 30% for postpartum depression [7-10]. The etiology of both pre- and postpartum depression still remains illusive. Various psycho-social and endocrine factors have been connected to both pre- and postpartum depression [2,3,11-14]. In addition, postpartum depression has been linked to a variety of endocrine root causes- especially postpartum thyroid dysfunction [15-17]. Antenatal depression has also been considered to increase the risk for postnatal depression [11,3]. This paper presents our findings on the prevalence of pre- and postpartum depression and risk factors responsible in a cohort of pregnant Afro-Jamaican women.

## Methods

One hundred and forty (140) healthy Afro-Jamaican pregnant women attending antenatal clinic in the Department of Obstetrics, Gynecology and Child Health, University Hospital of the West Indies, Jamaica (WI) during the period May 2000 to February 2001 consented to participate in this study. Women with a history of thyroid disease, any medical illness, depression or substance abuse were excluded. The mean age was 27 years, parity varied from primigravidae to 4 live births and the mean length of gestation was 8 weeks at booking. A relatively large number of these mothers (67) had to be excluded (45 failed to keep their antenatal appointments, 5 had miscarriages, 4 had initial thyroid dysfunction, 7 had premature deliveries, 1 developed severe hypertension during pregnancy and 5 were delivered by cesarean section). Thus a total of 73 pregnant women completed this study.

This study was duly approved by the UWI/UHWI Ethics Committee.

### Depression assessment

Each mother was given a questionnaire at booking to obtain a clinical profile and relevant demographic data. The Zung self-rating depression scale (SDS) questionnaire was administered at 28 weeks gestation and at 6 weeks postpartum. A participant was considered to have no psychopathy if Zung was <50, minimal to mild depression if Zung score was 50–59, moderate to marked depression if Zung score was 60–69, and severe to extreme depression if Zung score was 70 and over. Depending on the depression status, the cohort was subdivided into four subgroups (i) women depressed during prepartum only (ii) women depressed prepartum and who continued to be depressed postpartum (iii) women depressed postpartum only and (iv) women showing no signs of depression in either period.

### Hormonal profile

Blood samples were collected at 8, 28, 35 weeks of gestation and at 1 day and six weeks after childbirth. Serum total thyroxine (TT_4_), free triiodothyronine (FT_3_), thyrotropin (TSH) were determined using standard radioimmunoassay kits (Diagnostic Products Corporation, Los Angeles, California, USA). The sensitivity value for each assay was 0.25 pg/dl; 0.2 pg/ml; and 0.03 :IU/ml, respectively. All samples for each test were assayed in the same batch.

### Statistical analysis

Data are expressed as means ± SE or counts as appropriate. The data were analyzed by repeated measures analysis of variance (RMANOVA) with the between group factor being the four subgroups (i) women depressed during prepartum only (ii) women depressed prepartum and who continued to be depressed postpartum (iii) women depressed postpartum only and (iv) women showing no signs of depression in either period and the measurements done over time (8 wks 28 wks 35 wks 1 day postpartum and 6 wks postpartum) as the repeated measures factor. In analyses where there were significant interactions between the group factor and the repeated measures factor, we compared differences between the depression categories at each experiment and differences between the means of measured variables within each clinical category at each experiment, by Tukey method. In these comparisons the error term was the root mean square error from the RMANOVA analysis with its associated degrees of freedom.

The Zung self-rating depression scale (SDS) was used to classify participants into depression categories. For this analysis these categories were treated as an ordinal scale. To assess effects of changing thyroid hormones on the odds of changing depression categories postpartum we used only the measurements done at 28 wk prepartum and 6 weeks postpartum. We assessed the odds of being in a particular depressed category in the postpartum period for participants using an ordinal logistic model adjusting for prepartum depression category and differences in thyroid hormones level (6 wk postpartum – wk 28 prepartum values) as covariate. Inferential tests were considered statistically significant if p < 0.05 (two tail).

Data analysis was performed using Stata version8 for Windows (Statacorp, College station, TX).

## Results

### Pre- and postpartum depression

Self-rating depression scale administered to 73 women indicated that 41 women (56.16%) were having depression at 28 weeks prepartum. Out of these 41, 23 (31.5%) had mild, 13 (17.8%) had moderate and 5 (6.9%) had severe depression. Out of these 41, only 18 (24.66%) women were found to be depressed at 6 weeks postpartum. Remaining 23 (31.5%) women recovered anywhere between 28 weeks prepartum and 6 weeks postpartum period. The data further indicated that 4 out of the 5 women who were severely depressed at 28 weeks prepartum were only moderately depressed at 6 weeks postpartum; and the remaining had no depression. In addition, 7 (9.59%) different women were found to be depressed only at 6 weeks postpartum. Therefore, a total of 25 (34.25%) women suffered mild to marked depression during the postpartum period (Table 1).

**Table 1:** Results of Zung self-rating depression scale (SDS) questionnaire administered to 73 women at 28 weeks of gestation and at 6 weeks postpartum.

**SDS Index**	**Equivalent Clinical Global Impression**	**28 weeks Gestation Number of Cases**	**6 weeks Postpartum Number of cases**
**Below 50**	Normal range, no psychopathology	32 (43.8%)	48 (65.75%)
**50–59**	Presence of minimal to mild depression	23 (31.5%)	18 (24.66%)
**60–69**	Presence of moderate to marked depression	13 (17.8%)	7 (9.59%)
**70 and over**	Presence of severe to extreme depression	5 (6.9%)	None

### Demography and depression

Analysis of the data on marital status demonstrated that 31 (75.6%) out of 41 women who were depressed at 28 weeks prepartum were single or legally not married. 17 (94.4%) out of 18 women who continued to have depression at 6 weeks postpartum were also single. Similarly, 5 (71.4%) out of 7 women who suffered depression in postpartum period only, were single. Age, parity, miscarriages and employment status was not associated with depression (Table 2).

**Table 2:** Demographic characteristics by depression groups

**Variables**	**Never Depressed ** **N = 25**	**Prepartum Depression only ** **N = 23**	**Prepartum & Postpartum Depression ** **N = 18**	**Postpartum Depression only ** **N = 7**
**Mean Age (Yrs)**	27	27	25.9	28.4
**Single**	14(56%)	14(61%)	17(94%)	5 (71%)
**Primigravidae**	13 (52%)	11 (48%)	9 (50%)	3 (43%)
**Multiparous**	12 (48%)	12 (52%)	9 (50%)	4 (57%)
**Employed**	15 (60%)	15 (65%)	8 (44%)	6 (86%)
**Unemployed**	10 (40%)	8 (35%)	10 (56%)	1 (14%)

### Thyroid status and depression

**Figure 1:**
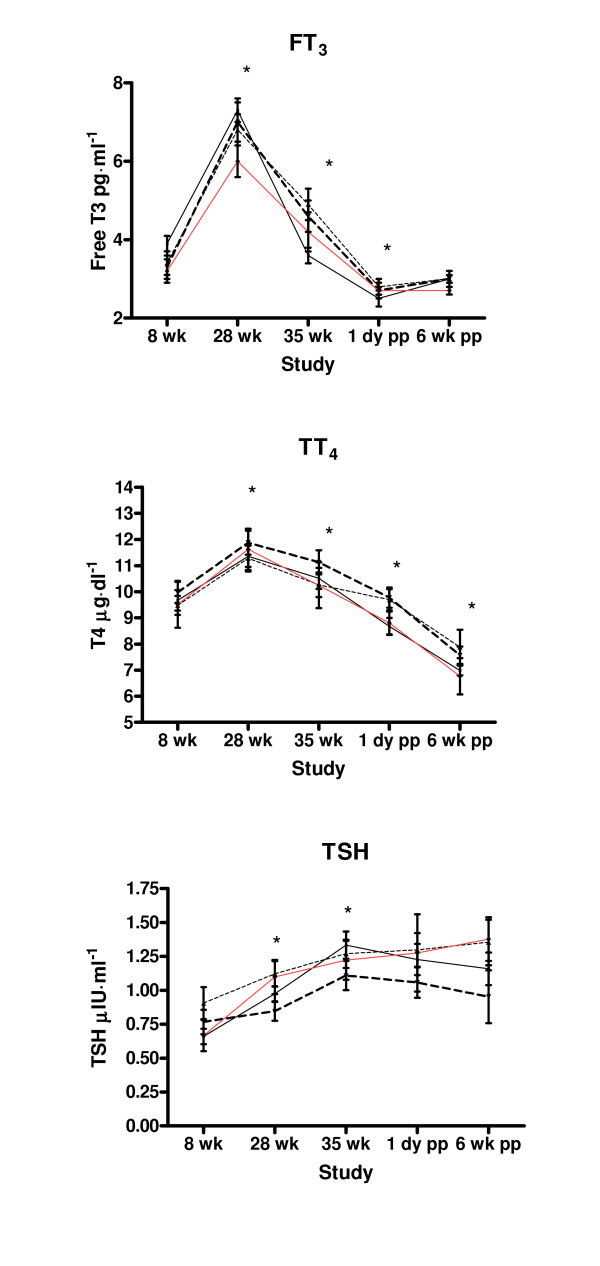
Thyroid hormones by depression groups. * Significantly different from previous values p < 0.05.

There were significant changes in the mean thyroid hormones concentrations over time (Fig 1). For both FT_3 _and TT_4_, mean values increased from week 8 to week 28 prepartum (p < 0.05) and then declined at the 35^th ^week (p < 0.05 compared with week 28) and 1 day post delivery study (p < 0.05 compared with week 35). There was a small increase in the mean FT_3 _values at 6 weeks postpartum compared to the 1 day postpartum values but this was not statistically significant. In contrast, the mean values for TT_4 _decreased further compared to the 1 day postpartum values (p < 0.05). The mean values for TSH increased significantly from week 8 through week 35. The mean values at 1 day postpartum and 6 week postpartum were not significantly different from the 35 week values. For FT_3_, TT_4 _and TSH there were no significant between group differences in concentrations. However there was a significant group by study interaction for FT_3_. At weeks 8 and 28 prepartum, the women who were never depressed tended to have higher mean FT_3 _values compared to women who were depressed. At week 35, the mean values for FT_3 _of women who were never depressed were significantly lower (p < 0.05) than mean FT_3 _values for women who were depressed prepartum.

There were no significant relationships between thyroid hormone concentrations and Zung scores at 28 weeks or at 6 week post partum. The major determinants of postpartum depression were moderate and severe prepartum depression and change in TT_4 _hormone concentrations (Table 3). Thus the odds of being depressed postpartum increased by factors of 4.8 in the moderately depressed category and 221 in the severely depressed category relative to not being depressed respectively adjusting for changes in TT_4_. For every unit increase in the magnitude of the difference in mean TT_4 _values between week 28 prepartum and 6 week postpartum the odds of being more depressed increases by a factor of 0.75 (p < 0.003).

**Table 3:** Prepartum determinants of a more severe depression post partum category relative to less severe depression postpartum category

**Prepartum Depression Category**	Factor change in odds*	**p value**
**Mild**	1.7	ns
**Moderate**	4.8	<0.002
**Severe**	221	<0.001
**Difference in T4 values**	0.75	<0.003

* Relative to not depressed category

## Discussion

The study demonstrated a higher prevalence rate of 56% maternal depression at 28 weeks prepartum as compared to 34 % at 6 weeks postpartum confirming that depression during pregnancy is as common as after delivery [1]. Our prevalence rates for both pre- and postpartum depression are higher than the rates reported earlier [1-3,7,8,10]. This may be attributed to the fact that greater percentages of women during both periods suffered from minimal to mild depression only, a score that might have been ignored by previous authors, who may have regarded it as clinically non-significant depression. Further analysis of the data on postpartum depression showed that prepartum depression was the major risk factor for postpartum depression [3,18].

Though the postnatal depression was observed only at 6 weeks postpartum, it is possible that the onset could have been within the first week after the delivery as reported earlier [9].

This study revealed that single mothers are more prone to depression both during pre- and postpartum periods. This can be interpreted in two ways (i) either that being single implies lack of a stable supporting union which influences depression and/or (ii) there is a predominance of single mothers in Jamaica (50 of the 73 mothers in this study were single). In both circumstances, whatever the cause of depression, the majority of subjects would be single. Employment, parity and the previous miscarriages did not seem to have any effect in producing depression.

In the sub-group of women representing 9.6% of the cohort, who developed depression in the postpartum period, relative hypothyroidism was observed during the late gestation and early postpartum periods. In addition, changes in mean TT_4 _levels were significantly related to postpartum depression. This finding is supported by previous studies [15-17] that suggest that postpartum thyroid dysfunction may be responsible for postpartum depression. However, this is in contrast to the study by Lucas et al [19] that has reported no link between postpartum thyroid dysfunction and postpartum depression.

## Conclusion

The results of our study limited only to corporate area, suggested almost equally high incidence of depression in single Jamaican mothers both during pre- and postpartum periods. In addition, relative hypothyroidism developed between late gestation and postpartum period could have been responsible for postpartum depression in a sub-group of mothers. In the light of the results, it is suggested that women who develop depression during pregnancy should be monitored for thyroid functions and social support be provided to single mothers to avoid the risk of postpartum depression. A wider cross- sectional study in Jamaica is further needed to confirm these results.

## Competing interests

The author(s) declare that they have no competing interests.

## Authors' contributions

**JW **M.Phil. Student who carried out this work.

**OP **chief supervisor, conceived and contributed to study design, reviewed the statistical analysis, interpretation of results and preparation of manuscript.

**SK **provided access to the subjects in the hospital, supervisory committee member, provided input on pregnancy matters.

## Pre-publication history

The pre-publication history for this paper can be accessed here:


